# Editorial: Abiotic stress combination: improving resilience to develop climate-smart crops

**DOI:** 10.3389/fpls.2026.1928281

**Published:** 2026-08-31

**Authors:** Hassan Iqbal, Chen Yaning, Muhammad Waqas, Syed Turab Raza, Muhammad Shareef, Christoph Martin Geilfus

**Affiliations:** 1 Xinjiang Institute of Ecology and Geography, Chinese Academy of Sciences, Ürümqi, Xinjiang, China; 2 Xinjiang Laboratory of Lake Environment and Resources in Arid Zone, College of Geographic Science and Tourism, Xinjiang Normal University, Urumqi, China; 3 Yunnan Key Laboratory of Meteorological Disasters and Climate Resources in the Greater Mekong Subregion, School of Earth Sciences, Yunnan University, Kunming, Yunnan, China; 4 Department of Botany, University of Narowal, Narowal, Punjab, Pakistan; 5 Department of Soil Science and Plant Nutrition, Hochschule Geisenheim University, Geisenheim, Germany

**Keywords:** agronomic interventions, climate-resilient agriculture, climate-smart crops, combined abiotic stress, crop resilience, genotype × environment interaction, non-additive stress responses, plant stress physiology

## Introduction

Climate change is intensifying the frequency, duration, and overlap of drought, salinity, heat, cold, flooding, and nutrient-related constraints, making combined and sequential abiotic stresses a defining challenge for crop productivity and food security. Against this backdrop, our Research Topic, *Abiotic Stress Combination: Improving Resilience to Develop Climate-Smart Crops*, was conceived to move beyond the traditional single-stress framework and instead highlight how plants integrate hormonal, metabolic, physiological, and molecular responses under complex environments. The collection was built around a central premise: progress toward climate-smart crops will depend not only on identifying stress-responsive traits, but on understanding how those traits interact across stress combinations, developmental stages, and production systems.

**Figure 1:**
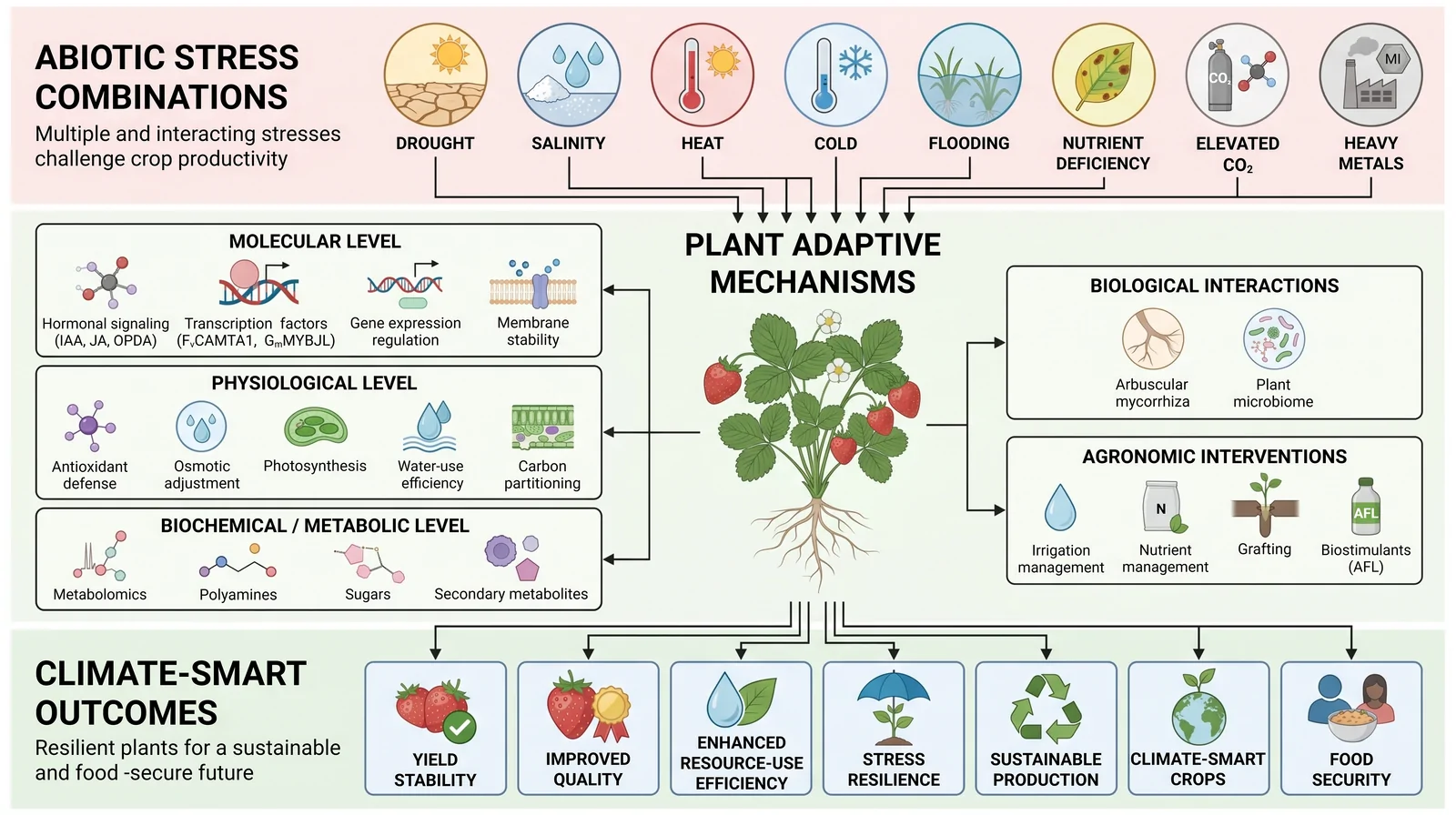
Schematic overview of the multiscale adaptive mechanisms, biological interactions, and agronomic interventions that collectively enhance resilience to abiotic stress combinations and support climate-smart crop development.

A major strength of this Research Topic is its breadth across biological scales and experimental contexts. The published articles span mechanistic studies in model and non-model species, physiology and management oriented work in field-relevant systems, quantitative genetic analyses, beneficial plant-microbe interactions and broader review articles that connect stress biology with breeding and restoration (**Table 1**). Together, these contributions demonstrate that crop resilience emerges from interactions among molecular regulation, genotype, management, and environmental complexity. Rather than viewing abiotic stresses as independent factors, this Research Topic demonstrates that plant responses to combined abiotic stresses are often non-additive and strongly context-dependent, varying with genotype, developmental stage, and management conditions. The Research Topic therefore argues that future crop-resilience research must move beyond single-stress descriptions and more firmly connect mechanistic insight with field validation, breeding, and agronomic innovation. **Figure 1** conceptually integrates the multiscale adaptive mechanisms, biological interactions, and agronomic interventions highlighted across the Research Topic, illustrating how these interconnected processes collectively contribute to climate-smart crop resilience.

**Table 1:** Overview of the contributions included in the Research Topic and their translational relevance.

Crop/species	Stress combination/condition	Main approach	Key translational relevance	Study
Rice	Saline-alkali stress	Bio-stimulant application	Sustainable saline soil management	Xu et al.
Zinnia elegans	Water × nitrogen interaction under cold-arid conditions	Physiological and agronomic analyses	Optimized water and nutrient management for sustainable ornamental production	Xu et al.
Finger millet	Salinity × irrigation regime under desert conditions	Genotype screening	Stress-resilient germplasm for cultivation in saline and water-limited environments	Talabi et al.
*Limonium* spp.	Salinity stress	Hormonal priming	Highlights hormonal priming as a practical strategy to enhance salinity tolerance	Vashishta et al.
*Arabidopsis thaliana*	Elevated CO_2_ × wounding	Signalling pathway analysis	Advances understanding of stress signalling under future atmospheric scenarios	Redl et al.
*Fraxinus velutina*	Salinity stress	Functional genomics	Identifies candidate genes for engineering salt-tolerant crops and trees	Yan et al.
Watermelon	Water limitation under arid conditions	Irrigation management	Strategies to improve water-use efficiency and fruit quality under water scarcity	Liu et al.
Grapevine	Arid conditions	Grafting physiology	Role of rootstock selection in enhancing stress resilience and fruit quality	Krishankumar et al.
*Miscanthus* hybrid	Drought × zinc stress	Multi-omics integration	Reveals multi-level adaptive mechanisms for breeding resilient bioenergy crops	Islam et al.
Soybean	Developmental stress/leaf senescence	Functional genomics	Transcriptional regulation associated with stress adaptation and productivity	Bao et al.
*Brassica rapa*	Cold stress	Membrane physiology	Identifies membrane-associated mechanisms underlying cold tolerance	Dong et al.
*Medicago truncatula*	Flooding × mycorrhiza	Plant–microbe interactions	Microbiome-assisted resilience	Rizi et al.
Rice	Reproductive-stage drought	QTL mapping	genomic resources for breeding drought-resilient rice cultivars	Venkateshwarlu et al.
Common bean	Moisture restriction during pod filling	Gene expression profiling	Improves understanding of carbon allocation mechanisms under drought	Morales-Elias et al.
Soybean	Drought stress	Comprehensive review	Provides an integrated framework for next-generation drought-resilient breeding	Burner et al.
*Apocynum* spp.	Drought × salinity	Comprehensive review	Establishes *Apocynum* as a model species for climate-smart dryland restoration	Shoukat et al.
Alfalfa	Salinity, drought, heavy metals	Comprehensive review	Targets for forage crop improvement	Daud et al.

## Molecular and regulatory networks underlying stress acclimation

Several contributions emphasize the central role of signaling and regulatory pathways in stress acclimation. Hormonal signaling, transcriptional regulation, and membrane-associated processes emerge repeatedly as key determinants of plant performance under stress combinations. Vashishta et al. demonstrated that priming with indole-3-acetic acid in *Limonium* enhanced salinity tolerance through coordinated modulation of antioxidant defenses, stress-responsive gene expression, and physiological performance, highlighting the translational potential of priming-based strategies for resilience under saline environments. Likewise, investigation in *Arabidopsis* revealed that interaction between CYP20–3 and oxophytodienoic acid flux under elevated CO_2_ conditions influence jasmonate biosynthesis Redl et al., illustrating how future atmospheric environments may reshape stress signaling pathways. and stress signaling.

Additional mechanistic studies further underscore the complexity of stress signaling networks. Yan et al. functionally characterized *FvCAMTA1* in *Fraxinus velutina* and demonstrated the role of calcium-mediated transcriptional regulation in salt stress adaptation. Likewise, Dong et al. showed that membrane rigidification influences *BrAFP1* and contributes to cold tolerance in Brassica rapa, highlighting the importance of membrane-associated processes in environmental adaptation. Bao et al. characterized the soybean *MYB* transcription factor *GmMYBLJ*, providing new insights into transcriptional regulation associated with leaf senescence, a developmental process closely linked to stress adaptation and productivity. Together, these findings illustrate that resilience emerges from coordinated regulatory networks rather than isolated signaling pathways, reinforcing the need for systems-level approaches to understanding combined-stress adaptation.

## Integrating physiology, management, and developmental plasticity for resilience

A recurring conceptual message emerging from this Research Topic is that crop resilience is an emergent systems-level property arising from interactions among genotype, environment, developmental stage, and crop management. Several contributions demonstrate that stress tolerance is shaped not only by intrinsic genetic potential but also by agronomic interventions and local production conditions.

Talabi et al. examined the differential responses of finger millet accessions to contrasting saline-water levels and irrigation regimes under desert conditions, providing an excellent example of genotype × environment interactions under combined stress scenarios. The substantial variability observed among accessions highlights the importance of exploiting genetic diversity for crop improvement in water-limited and saline agroecosystems. Complementing these findings, Xu et al. demonstrated that integrated water and nitrogen management in *Zinnia* grown under cold and arid conditions substantially influenced soil characteristics, physiological metabolism, and ornamental quality. These findings emphasize that resource management strategies can significantly modulate plant responses to environmental stress.

Studies by Liu et al. on watermelon and Krishankumar et al. on grapevine further extend this perspective by illustrating how management interventions can improve both productivity and quality under arid conditions. Optimization of mulched drip irrigation enhanced water-use efficiency and fruit quality in watermelon, whereas different scion-rootstock combinations significantly influenced sugar accumulation, polyamine metabolism, antioxidant capacity, and oxidative damage in grafted grapevines. These contributions strongly support the notion that climate-smart crop development must integrate stress physiology with practical, system-level crop management approaches capable of maximizing resource-use efficiency under increasingly constrained environmental conditions.

An additional dimension emerging from this Research Topic is the growing importance of integrative and multi-scale approaches for understanding plant responses to stress combinations. Traditional physiological assessments remain indispensable; however, several studies demonstrate that combining physiological, biochemical, and molecular analyses provides a more comprehensive understanding of resilience mechanisms. For instance, Islam et al. studied a novel *Miscanthus* hybrid exposed to combined drought and zinc stress and showed that coordinated adjustments in metabolism, antioxidant defense, and resource allocation underpin tolerance to multiple simultaneous stresses. This illustrates how combining multiple analytical platforms can reveal emergent resilience mechanisms that remain hidden when individual stress responses are examined in isolation. Capturing these interactions across multiple organizational levels will be essential for identifying robust traits suitable for future crop improvement programs.

The Research Topic also highlights the importance of developmental stage-specific responses to environmental stress. Crop sensitivity often varies substantially across developmental stages, and failure to consider this temporal dimension may constrain breeding progress. Venkateshwarlu et al. mapped genomic regions associated with reproductive-stage drought tolerance in rice, directly addressing one of the most vulnerable stages of crop development. Because drought during reproductive growth can severely reduce grain yield, the identification of genomic regions associated with tolerance offers valuable opportunities for marker-assisted selection and genomic breeding. Similarly, Morales-Elias et al. demonstrated that members of the sucrose synthase gene family in common bean are differentially regulated during pod filling under moisture restriction, illustrating how carbon metabolism and assimilate partitioning are reprogrammed during critical developmental stages. Together, these studies emphasize that future breeding programs should account not only for stress intensity and type, but also for developmental timing, phenological plasticity, and stage-specific adaptive responses.

Another important contribution of this Research Topic is the recognition that beneficial plant-microbe interactions can significantly influence resilience under complex environmental conditions. Safavi-Rizi et al. examined the impact of arbuscular mycorrhizal colonization on flooding responses in *Medicago truncatula*, demonstrating how symbiotic interactions can modify plant adaptation to water-related stress. As climate change increases the frequency of flooding and waterlogging events in many regions, understanding the role of microbial partners in mitigating stress effects becomes increasingly relevant. Plant-associated microbiomes are now recognized as critical components of plant adaptive capacity, influencing nutrient acquisition, hormonal signaling, oxidative balance, and overall plant performance. Consequently, climate-smart crop development should increasingly incorporate microbiome-based approaches alongside conventional breeding and agronomic management strategies.

Xu et al. demonstrated that application of apple fermentation liquid improved growth and stress responses in rice grown under saline-alkali soil conditions, further illustrating the potential of sustainable interventions for enhancing crop resilience. The effectiveness this treatment highlights the promise of biostimulant-based strategies for maintaining productivity in degraded soils. Such environmentally friendly approaches may become increasingly important as agricultural systems face intensifying soil salinization and land degradation.

## Translational pathways toward climate-smart crop development

The review articles included in this Research Topic provide an essential bridge between mechanistic understanding and translational opportunity. The comprehensive review by Burner et al. on soybean drought tolerance synthesizes advances in genetics, metabolomics, remote sensing, and breeding, illustrating how future crop improvement will increasingly rely on integrating phenomics, genomics, and precision agriculture technologies. Shoukat et al. focusing on *Apocynum*, presents a multiscale perspective on combined drought and salinity stress, positioning this species as a valuable model for climate-smart dryland restoration and sustainable production systems. Meanwhile, Daud et al. expand the discussion to salinity, drought, and heavy metal toxicity in alfalfa, integrating physiological and molecular perspectives to identify future directions for stress resilience research in forage crops. Importantly, these reviews frame the Research Topic not simply as a series of independent studies, but as a roadmap for integrating stress biology, breeding innovation, ecosystem restoration, and resilience-oriented crop design.

Beyond their individual findings, the studies assembled in this Research Topic collectively define four conceptual principles that may guide future research on combined abiotic stress and climate-smart crop development. First, combined abiotic stresses generate emergent responses that frequently differ from those observed under individual stresses, underscoring the limitations of reductionist single-stress approaches. Second, stress resilience is highly context dependent, reflecting the dynamic interplay among developmental stage, genotype, environmental conditions, and crop management. Third, meaningful advances in crop improvement will require integrating mechanistic insights from molecular and physiological studies with breeding strategies, high-throughput phenotyping, and field-based validation. Finally, the Research Topic highlights that developing climate-smart crops is inherently a multidisciplinary challenge, requiring coordinated efforts across plant biology, agronomy, genetics, and environmental sciences to translate fundamental discoveries into resilient agricultural systems.

Despite this progress, important challenges remain. Combined-stress experiments still differ widely in species, developmental stage, stress intensity, and treatment timing, limiting cross-study comparability and reducing predictive power. Moreover, mechanistic interpretations should be made cautiously when not supported by agronomically meaningful endpoints such as yield stability, product quality, and field performance. Future work will therefore benefit from greater standardization, stronger field validation, and closer integration of physiology, omics, phenotyping, and breeding. Such advances will help ensure that candidate resilience traits are validated not only by stress-marker responses but also by their ability to sustain yield and crop quality under realistic field conditions.

Rapid advances in multi-omics technologies, high-throughput phenotyping, artificial intelligence, and machine learning provide unprecedented opportunities to accelerate climate-smart crop improvement. Integrating these emerging technologies with genomics-assisted breeding, precision agriculture, and systems biology will enable the identification of key regulatory networks, predictive resilience traits, and data-driven management strategies capable of enhancing crop adaptation under increasingly complex environmental conditions.

Overall, this Research Topic highlights the multidimensional nature of abiotic stress resilience and demonstrates that developing climate-smart crops requires coordinated advances in signaling biology, physiology, genomics, agronomy, and breeding. Collectively, the studies presented here show that resilience under combined abiotic stresses is not a fixed trait but a context-dependent outcome emerging from dynamic interactions among molecular regulation, developmental processes, environmental complexity, and crop management. Beyond summarizing recent advances, this Research Topic establishes a conceptual framework that encourages more realistic combined-stress experimentation, stronger interdisciplinary collaboration, and closer integration between mechanistic research, field validation, breeding, and agricultural application. Ultimately, the transition from describing plant stress responses to predicting and engineering crop performance under realistic environmental complexity will define the next generation of climate-smart agriculture.

